# Underfocus Laser Induced Ni Nanoparticles Embedded Metallic MoN Microrods as Patterned Electrode for Efficient Overall Water Splitting

**DOI:** 10.1002/advs.202105869

**Published:** 2022-02-03

**Authors:** Yuke Chen, Yijie Wang, Jiayuan Yu, Guowei Xiong, Hongsen Niu, Yang Li, Dehui Sun, Xiaoli Zhang, Hong Liu, Weijia Zhou

**Affiliations:** ^1^ Collaborative Innovation Center of Technology and Equipment for Biological Diagnosis and Therapy in Universities of Shandong Institute for Advanced Interdisciplinary Research (iAIR) University of Jinan Jinan 250022 P. R. China; ^2^ School of Information Science and Engineering Shandong Provincial Key Laboratory of Network Based Intelligent Computing University of Jinan Jinan 250022 P. R. China; ^3^ School of Materials Science and Engineering Zhengzhou University Zhengzhou 450001 P. R. China; ^4^ State Key Laboratory of Crystal Materials Shandong University Jinan 250100 P. R. China

**Keywords:** industrial water‐splitting electrolyzer, large current density, Ni/MoN microrod, superstability, underfocus laser heating

## Abstract

Transition metal nitrides have shown large potential in industrial application for realization of the high active and large current density toward overall water splitting, a strategy to synthesize an inexpensive electrocatalyst consisting of Ni nanoparticles embedded metallic MoN microrods cultured on roughened nickel sheet (Ni/MoN/rNS) through underfocus laser heating on NiMoO_4_·xH_2_O under NH_3_ atmosphere is posited. The proposed laser preparation mechanism of infocus and underfocus modes confirms that the laser induced stress and local high temperature controllably and rapidly prepared the patterned Ni/MoN/rNS electrodes in large size. The designed Ni/MoN/rNS presents outstanding catalytic performance for hydrogen evolution reaction (HER) with a low overpotential of 67 mV to deliver a current density of 10 mA cm^−2^ and for the oxygen evolution reaction (OER) with a small overpotential of 533 mV to deliver 200 mA cm^−2^. Density functional theory (DFT) calculations and Kelvin probe force microscopy (KPFM) further verify that the constructed interface of Ni/MoN with small hydrogen absorption Gibbs free energy (Δ*G*_H*_) (−0.19 eV) and similar electrical conductivity between Ni and metallic MoN, which can explain the high intrinsic catalytic activity of Ni/MoN. Further, the constructed two‐electrode system (−) Ni/MoN/rNS||Ni/MoN/rNS (+) is employed in an industrial water‐splitting electrolyzer (460 mA cm^−2^ for 120 h), being superior to the performance of commercial nickel electrode.

## Graphical abstract

The Ni nanoparticles embedded metallic MoN microrods are uniformly fabricated on rNS (Ni/MoN/rNS) with “star” pattern by underfocus laser heating of NiMoO_4_·xH_2_O microrods under NH_3_ atmosphere. The Ni/MoN/rNS||Ni/MoN/rNS is employed in an industrial water‐splitting electrolyzer, being superior to the performance of commercial nickel electrode.

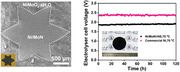

## 1 Introduction

With the burning of fossil fuels and high volume of consumption of non‐renewable energy, it was urgent to address a series of energy shortage and environmental pollution problems.^[^ ^1^ ^]^ In this regard, the development and utilization of clean and renewable energies had become the central node. As a clean energy, hydrogen (H_2_) could not only be produced from water with no polluting gases during the combustion process, but it was also easily manufactured by electrolysis of water, as it could be driven by some renewable energy sources, like wind, solar, and water energy.^[^ ^2^ ^]^ Hydrogen generated by electrolysis of water was considered as one of the most efficient and simple methods.^[^ ^3^ ^]^ High‐efficiency and ideal catalysts often required lower HER overpotential to maintain large current density.^[^ ^4^ ^]^ A large number of studies had shown that precious metal‐based catalysts (i.e., Pt, RuO_2_, and IrO_2_) possessed great activity in hydrogen production. Nevertheless, its scarcity and high price conformed to its limitation in large‐scale industrial productions.^[^ ^5^ ^]^ Therefore, it was urgent to exploit cost‐effective, highly active, and stable electrocatalyst for electrolysis of water.

Recently, non‐noble metal‐based catalysts had been identified as the alternative of noble metal‐based one to settle down water splitting, such as transition metal alloys,^[^ ^6^ ^]^ nitrides,^[^ ^7^ ^]^ sulfides,^[^ ^8^ ^]^ carbides,^[^ ^9^ ^]^ and phosphides.^[^ ^10^ ^]^ Especially, molybdenum nitride possessed metallic conductivity and excellent mechanical robustness by virtue of their unique structure, where nitrogen atoms occupied interstitial positions in the metal lattice. It was an important factor for electrocatalysis.^[^ ^11^ ^]^ Besides, the introduction of metal‐nitrogen bond would expand the original lattice while at the same time reduce the d‐band contraction.^[^ ^12^ ^]^ For example, MoN possessed similar catalytic activity in contrast to noble metals, which was credit to the d‐band contraction and higher density of states near the metal Fermi level. According to “volcano plot,” there was a certain correlation between the HER activity and hydrogen–metal bond strength.^[^ ^13^ ^]^ Therefore, the MoN with strong hydrogen binding energy could combine with a weaker metal like Ni to achieve a relatively moderate value. Based on the aforementioned concepts, it was worthy of achieving the outstanding HER activity by virtue of the synergistic effect between Ni and MoN electrocatalysts.^[^ ^14^ ^]^ The synthesis of molybdenum nitride usually required the harsh conditions, such as the high temperature and time‐consuming calcination in the NH_3_ atmosphere.^[^ ^15^ ^]^ For example, Zhou et al.^[^ ^16^ ^]^ synthesized two‐dimensional metallic MoN film under the temperature of 700 °C by a scalable salt‐templating method. Although the furnace device was commonly used as traditional radiation heating method,^[^ ^17^ ^]^ it required a large amount of reaction atmosphere and high energy consumption to reach the high temperatures.^[^ ^18^ ^]^ Therefore, it was particularly important to develop an efficient synthesis method for nickel and MoN with specific nanostructures, which could directly and rapidly synthesize micro/nanomaterials at low temperature, and replace the traditional high energy consuming method.

Based on the waves and particle properties of laser, laser ablation,^[^ ^19^ ^]^ laser patterning design,^[^ ^20^ ^]^ and local thermal effect^[^ ^21^ ^]^ had been found extended application in controlled manipulation of electrode materials, such as grain boundaries‐rich Ru nanoparticle,^[^ ^22^ ^]^ stacking faults Ag nanoparticle,^[^ ^23^ ^]^ and RuAu nanoalloy.^[^ ^24^ ^]^ As for laser ablation and laser patterning, the structure of catalysts was damaged because of the ultra‐high concentrated energy density,^[^ ^25^ ^]^ and created metastable suprananoparticles with abundant reaction sites.^[^ ^26^ ^]^ Laser heating with high local thermal effect was adopted to synthesize the carbon materials^[^ ^27^ ^]^ and achieve element doping.^[^ ^28^ ^]^ The laser heating directly acted on the material with small amount of heat loss while the temperature could be tuned by output power and underfocus degree. Tour's group reported three‐dimensional (3D) porous graphene by a one‐step laser‐scribing process on commercial polyimide film with the advantages of patterned design.^[^ ^29^ ^]^ The laser heating process greatly reduced the reaction time and simplified the manufacturing process, which possessed advantages of high controllability and low energy consumption. Although there were lots of advanced works reported the metal‐carbon based material by CO_2_ laser burning of metal‐organic complexes, few reports had probed into pure metal nitrides produced by underfocus laser‐heating.

Herein, we demonstrated a general and facile laser heating method to synthesize transition metal nitrides, carbides, sulfides and alloy, which could provide high local energy and patterned design. By controlling the underfocus length (UL), the different reaction temperature and pressure were achieved to synthesized Ni nanoparticles embedded metallic MoN microrods derived from NiMoO_4_·xH_2_O microrods under NH_3_ atmosphere at low environment temperature. The obtained Ni/MoN microrods cultured on roughened nickel sheet (Ni/MoN/rNS) showed excellent catalytic performance for HER (640 mA cm^−2^ at −0.87 V vs RHE for 12 h), OER (650 mA cm^−2^ at 2.19 V vs RHE for 10 h) and overall water splitting (500 mA cm^−2^ with 2.8 V for 30 h). More importantly, the large‐scaled Ni/MoN/rNS prepared by laser heating also showed excellent activity in industrial water splitting equipment (460 mA cm^−2^ for 120 h), which proved the potential of laser heating method and industrial applications of Ni/MoN/rNS catalyst for overall water splitting.

## 2 Results and Discussion

**Figure 1:**
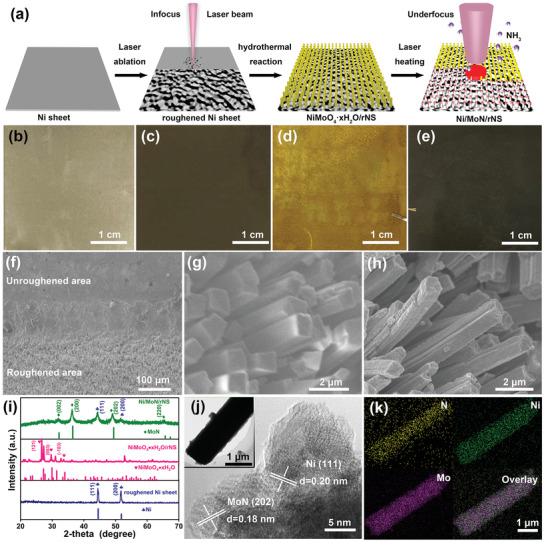
a) Synthetic scheme of Ni/MoN/rNS. Photographs of b) Ni sheet, c) rNS, d) NiMoO_4_·xH_2_O/rNS, and e) Ni/MoN/rNS. SEM images of f) smooth area and rNS, g) NiMoO_4_·xH_2_O/rNS, and h) Ni/MoN/rNS. i) XRD patterns of rNS, NiMoO_4_·xH_2_O/rNS, and Ni/MoN/rNS. j) High resolution transmission electron microscopy (HRTEM) image (inset: the TEM image of Ni/MoN microrod) and k) elemental mapping of Ni/MoN microrod.

The manufacturing and preparation process of the Ni/MoN/rNS contained three steps, as clarified in **Figure** **1**a. First, the Ni sheet (Figure 1b) with smooth surface was roughened by infocus laser ablation (laser scan parameter: line spacing of 5 µm, output power of 12.5 W, sweep rate of 1000 mm s^−1^, Figure 1c). The color of Ni sheet was changed from gray to dark after the roughening process. Scanning electron microscopy (SEM) image (Figure 1f) and X‐ray diffractometer (XRD) pattern (Figure 1i) confirmed that Ni microparticles were only anchored at roughened area on the surface of roughened nickel sheet (rNS). Second, the NiMoO_4_·xH_2_O microrods with yellow color were grown on the rNS by hydrothermal method (Figure 1d). SEM image in Figure 1g outlined that NiMoO_4_·xH_2_O microrods were 1–2 µm in diameter and ≈10 µm in length. The XRD pattern in Figure 1i illustrated that the characteristic diffraction peaks of the NiMoO_4_·xH_2_O at 27.20°, 30.23°, and 34.21° were corresponded to the (121), (003), and (−103) of the NiMoO_4_·xH_2_O (PDF card No. 04‐017‐0338).^[^ ^30^ ^]^ Third, the NiMoO_4_·xH_2_O/rNS was put into the laser reaction chamber (Figure S1, Supporting Information) under NH_3_ atmosphere, which was to synthesize Ni/MoN/rNS with size of 4 × 4 cm^2^ by underfocus laser heating method within 5 min. The color of sample was changed from yellow to black (Figure 1e). SEM image in Figure 1h indicated that the microrods became rough, but the overall morphology was well maintained after laser heating. The XRD pattern in Figure 1i illustrated that the diffraction peaks at 31.89°, 36.20°, 49.01° were corresponded to (002), (200), (202) plane of MoN (PDF card No. 25‐1367) and 44.50° (111), 51.84° (200) were characteristic peaks of Ni (PDF card No. 04‐0850). The atomic ratio of Ni:Mo of the Ni/MoN/rNS was further determined to be 1:0.4 by inductively coupled plasma‐atomic emission spectroscopy (ICP‐AES) analysis (Table S1, Supporting Information). Transmission electron microscopy (TEM) was adopted further to characterize the crystalline structure of Ni/MoN microrod. Figure 1j depicted that a lattice spacing was 0.18 nm, belonged to (202) of MoN and a lattice distance of 0.20 nm corresponded to (111) of Ni. Energy dispersive spectroscopy (EDS) analysis verified uniform dispersed of Ni, Mo, and N elements in Ni/MoN microrod, indicating the Ni and MoN were distributed in microrod homogeneously (Figure 1k).

To further investigate the chemical composition on surface of Ni/MoN/rNS and NiMoO_4_·xH_2_O/rNS, X‐ray photoelectron spectroscopic (XPS) measurements were displayed in Figure S2 (Supporting Information). The survey XPS all spectrum (Figure S2a, Supporting Information) verified the coexistence of Ni and Mo elements in both NiMoO_4_·xH_2_O/rNS and Ni/MoN/rNS. Yet N element was discovered merely in the Ni/MoN/rNS. Figure S2b (Supporting Information) presented high resolution XPS of Mo 3d, and the peaks located at 235.7 and 232.5 eV were belong to high oxidation states (Mo^4+^ and Mo^6+^),^[^ ^31^ ^]^ because the surface Mo was susceptible to oxidation when exposed to the air.^[^ ^32^ ^]^ As for Ni/MoN/rNS, the Mo^3+^ with peaks at 228.7 and 232.0 eV were verified, which was corresponded to the Mo–N in MoN. Figure S2c (Supporting Information) showed the XPS spectrum of Ni 2p in NiMoO_4_·xH_2_O/rNS and Ni/MoN/rNS, which could be divided into two main peaks, namely 855.7 eV (Ni 2p_3/2_) and 872.7 eV (Ni 2p_1/2_).^[^ ^33^ ^]^ For Ni/MoN/rNS, two additional peaks at 852.8 and 870.0 eV were corresponded to the Ni^0^ due to the occurrence of Ni particle. Lastly, for Ni/MoN/rNS, as shown in Figure S2d (Supporting Information), XPS peak of N 1s was detected at 397.7 eV, correlating with N—Mo bonding.^[^ ^13^, ^34^ ^]^ The above XPS results confirmed that Ni/MoN/rNS was successfully synthesized.

**Figure 2:**
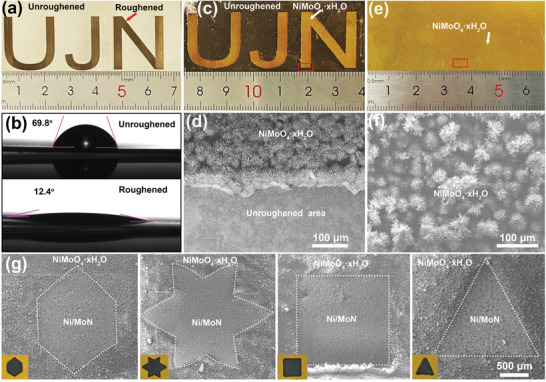
a) “UJN” pattern of rNS and b) contact angle images for droplets water on Ni sheet and rNS. c) “UJN” pattern of NiMoO_4_·xH_2_O supported on rNS. d) SEM image of the red marked place in (c). e) Photograph and f) SEM image of NiMoO_4_·xH_2_O/rNS. g) SEM images and photographs of Ni/MoN/rNS with different shapes.

Due to the narrow line width of 5 µm and locally heated areas, the laser could selectively prepare microstructures on the specified surface of substrate. Roughened Ni sheet with an “UJN” pattern was produced by laser ablation in the infocus mode (**Figure** **2**a). This roughening process was able to make a superhydrophobic surface with water droplets, which exhibited contact angles of 12.4^o^, smaller than that of Ni sheet with smooth surface (69.8^o^, Figure 2b). Thanks to the rough surface and hydrophilicity, the nucleation and growth of NiMoO_4_·xH_2_O microrods selectively carried out on the roughened Ni sheet. After the hydrothermal reaction, the dense and uniform NiMoO_4_·xH_2_O microrods were selectively grown on rNS with “UJN” pattern (Figure 2c,d). No NiMoO_4_·xH_2_O microrods were observed on the unroughened area of Ni sheet. In addition, the NiMoO_4_·xH_2_O microrods could uniformly be grown on the roughened Ni sheet in large scale of 3 × 6.5 cm (Figures 2e,f and 1d). Except for the laser coarsening under focused mode, the selective laser heating under unfocused mode enabled the synthesis of patterned micromaterials. The different patterns of Ni/MoN/rNS with micrometer scale, like hexagon, hexagram, square, and triangle, were produced by virtue of underfocus laser heating on NiMoO_4_·xH_2_O/rNS (Figure 2g), confirming the high regional selectivity.

**Figure 3:**
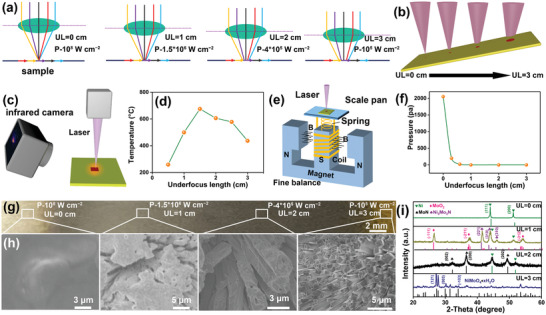
a,b) Schematic diagrams of different laser underfocus modes. c) Synthetic scheme of laser thermal image and d) according laser‐produced temperature. e) Synthetic scheme of laser‐induced pressure test and f) according laser‐induced pressure. g) Photograph, h) SEM images and i) XRD patterns of four points synthesized at different degree of underfocus laser with UL ranged from 0 to 3 cm with interval of 1 cm on the NiMoO_4_·xH_2_O/rNS.

In order to further explore the underfocus mode (thermal effect) and focused mode (ablation effect), as we could see in **Figure** **3**a,b, the different degree of underfocus laser could be achieved by tuning the UL from 0 to 3 cm, while the power density (P) could be tuned from 10^8^ to 10^5^ W cm^−2^. The relationship between the laser temperature and the UL from 0.5 to 3 cm with interval of 0.5 cm were testified in detail by thermal imager (Figure 3c,d and Figure S3, Supporting Information). The corresponding temperature gradually increased from 259.1, 499.8 to 675.3 °C upon the UL varying from 0.5, 1, to 1.5 cm, subsequent dropped to 606.3, 578.2, and 436.8 °C with the UL increased from 2, 2.5, to 3 cm. In addition, the relationship between the laser induced pressure and the UL from 0 to 3 cm were verified by self‐built pressure sensors, as shown in Figure 3e. In Figure 3f, the highest pressure was detected to be 2000 Pa in infocus mode with UL of 0 cm. It then fleetly dropped to 200 Pa (UL = 0.3 cm) and 0 Pa (UL > 0.6 cm). Therefore, the laser in infocus mode carried out the etching reaction, which produced the low temperature but large pressure. Conversely, if it worked out with heating reaction, it produced the high temperature but small pressure.

To explore the working mechanism of laser in different modes, the NiMoO_4_·xH_2_O/rNS rectangular sheet with size of 5 × 0.5 cm was tilted in the reactor with an angle of 45^o^ and treated by laser in a continuous increasing UL under NH_3_ atmosphere, as shown in Figure 3b,g. Four different positions on NiMoO_4_·xH_2_O/rNS were selected to perform SEM images in Figure 3h and XRD characterization in Figure 3i. The according color of NiMoO_4_·xH_2_O/rNS was altered gradually from yellow to black (Ni/MoN) and silver gray (Ni sheet) with UL from 3 to 0 cm. As it could be seen from the focus laser with ablation mode (UL = 0, P = 10^8^ W cm^−2^), the concentrated light spot completely etched the NiMoO_4_·xH_2_O microrods, soon afterwards the Ni substrate was exposed, as shown in Figure 3h. No NiMoO_4_·xH_2_O and MoN were detected by XRD pattern with only Ni sheet was remained, as shown in Figure 3i. When the UL was increased to 3 cm with the optical power density of 10^5^ W cm^−2^, the color, morphology, and crystal phase of NiMoO_4_·xH_2_O were not changed compared with that of present NiMoO_4_·xH_2_O (Figure 1d,g,i), due to the weak etching energy and low heating temperature (Figure 3g,i). Through optimizing the UL of laser (2 cm, 4 × 10^5^ W cm^−2^), the phase transformation from NiMoO_4_·xH_2_O to Ni/MoN was implemented for the sake of the high temperature and the microrod morphology was well maintained due to weak laser ablation.

**Figure 4:**
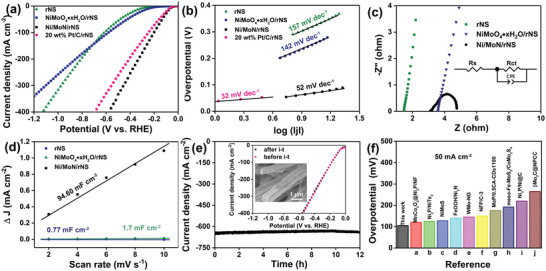
a) LSV curves and b) Tafel slopes of rNS, NiMoO_4_·xH_2_O/rNS, Ni/MoN/rNS, and 20 wt% Pt/C/rNS toward HER in 1 m KOH. c) Nyquist plots with the overpotential of 76 mV (inset: the equivalent circuit) and d) Electrochemical area of rNS, NiMoO_4_·xH_2_O/rNS, Ni/MoN/rNS. e) Long‐term stability of Ni/MoN/rNS toward HER (inset: The polarization curves of Ni/MoN/rNS before and after *i*–*t* testing and SEM image of Ni/MoN/rNS after *i*–*t* testing). f) Comparison of the required overpotential at a current density of 50 mA cm^−2^ for the Ni/MoN/rNS with reported state‐of‐the‐art catalysts.^[^ ^15^, ^35^ ^]^

The HER catalytic activities of rNS, NiMoO_4_·xH_2_O/rNS, Ni/MoN/rNS, and 20 wt% Pt/C/rNS were examined as the working electrode and performed by a typical three‐electrode electrolytic cell in 1 m KOH. The Ni/MoN/rNS prepared by laser heating displayed distinguished HER activity with a small overpotential of 67 mV at current density of 10 mA cm^−2^ (Figure **4**a), which was much better than those of rNS (316 mV) and NiMoO_4_·xH_2_O/rNS (248 mV), and close to that of 20 wt% Pt/C loaded on rNS (62 mV). Moreover, the Ni/MoN/rNS exhibited an overpotential of 514 mV at current density of 500 mA cm^−2^, smaller than that of Pt/C/rNS (545 mV). The 3D microrods array structure and high active materials of Ni and MoN microrod on rNS promoted electron transfer, hydrogen escape, and enabled the realization of high current densities for HER.

In order to explain the kinetic reaction mechanism of HER, Figure 4b illustrated the Tafel plots of samples, derived from the polarization curves in Figure 4a. Notably, the Ni/MoN/rNS exhibited Tafel slope of 52 mV dec^−1^, which was less than those of rNS (157 mV dec^−1^), NiMoO_4_·xH_2_O/rNS (142 mV dec^−1^) and was very approached to that of 20 wt% Pt/C/rNS (32 mV dec^−1^). Such small Tafel of Ni/MoN/rNS disclosed that electrocatalytic HER performance were determined by the Heyrovsky step. Moreover, the charge‐transfer resistance (R_ct_) of electrocatalyst were also detected by the electrochemical impedance spectroscopy (EIS) analysis with an overpotential of 76 mV, as shown in Figure 4c. Apparently, the resistance (R_s_) value of Ni/MoN/rNS (3.1 Ω) was close to that of rNS (1.68 Ω) and smaller than that of NiMoO_4_·xH_2_O/rNS (3.6 Ω), indicating the Ni/MoN had an excellent conductivity. Moreover, the R_ct_ of Ni/MoN/rNS was 0.8 Ω at 76 mV, far less than those of rNS (>500 Ω) and NiMoO_4_·xH_2_O/rNS (101 Ω), showing that the Ni/MoN/rNS possessed a fast electrocatalytic kinetic ability. Lastly, the R_ct_ values of the Ni/MoN/rNS decreased from 1 to 0.5 Ω with increased overpotentials from 56 to 76 mV (Figure S4, Supporting Information), indicating the faster charge transfer between Ni/MoN/rNS and electrolyte even at low overpotentials.^[^ ^36^ ^]^ Moreover, the electrochemical double‐layer capacitance (C_dl_) was performed to assess the electrochemically effective surface area (ECSA) of Ni/MoN/rNS, as shown in Figure 4d and Figure S5 (Supporting Information). It was observed that Ni/MoN/rNS presented a high C_dl_ value of 94.6 mF cm^−2^, which was larger than those of rNS (0.77 mF cm^−2^) and NiMoO_4_·xH_2_O/rNS (1.7 mF cm^−2^), implying the more catalytic active sites of Ni/MoN/rNS. After being revised by ECSA (Figure S6, Supporting Information), the calibrated current density of Ni/MoN/rNS was smaller than those of rNS and NiMoO_4_·xH_2_O/rNS. Nevertheless, Ni/MoN/rNS still expressed the smaller onset potential compared with rNS and NiMoO_4_·xH_2_O/rNS, indicating higher active for HER. Hence, the high active and more catalytic activity sites enabled the better HER performance.

Further, the current–time (*i*–*t*) testing of Ni/MoN/rNS was carried out to explore the stability of the catalyst. As shown in Figure 4e, the catalyst showed the stable and large current density of 640 mA cm^−2^ for 12 h at large overpotentials of 876 mV. Moreover, Linear sweep voltammetry (LSV) curves of before and after *i*–*t* testing showed unchanged current density, demonstrating the retained steady HER activity. Similarly, there were no changes of the morphology and structure of Ni/MoN/rNS (inset of Figure 4e) and XRD pattern (Figure S7, Supporting Information) after durability test, suggesting the outstanding durability. For comparing with others non‐noble‐metal electrocatalysts, an overview of overpotential for delivering 50 mA cm^−2^ in literature were collected in Figure 4f and Table S1 (Supporting Information).

**Figure 5:**
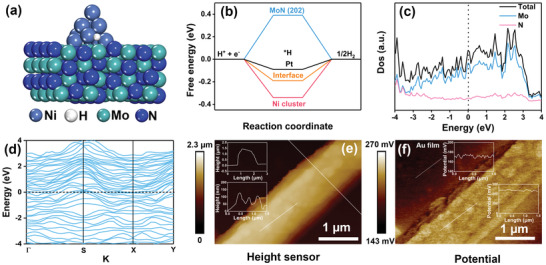
a) The optimized structure of Ni cluster supported on the MoN (202). b) The calculated hydrogen adsorption free energy. c) The density of state (Dos) and d) band structure of MoN. e) AFM image and f) potential image of Ni/MoN microrod (inset: the corresponding height profile and potential profile, respectively).

To study the HER intrinsic catalytic ability of Ni/MoN, the MoN microrods were constructed by acid‐etching treatment of Ni/MoN microrods, due to the unstable metallic Ni in acid (Figure S8, Supporting Information). In addition, the porous Ni nanorods were synthesized by calcination of NiC_2_O_4_·2H_2_O nanorods (Figure S9, Supporting Information). The Ni/MoN catalyst exhibited the lowest overpotential of 110 mV to deliver a current density of 10 mA cm^−2^, which was lower than that of MoN (270 mV) and Ni (230 mV), as shown in Figure S10 (Supporting Information). Therefore, the excellent HER intrinsic catalytic ability of Ni/MoN was contributed to the synergetic effect between Ni and MoN. To deeply identify the synergistic effect Ni and MoN, DFT calculation was employed to calculate the ΔG_H*_ of Ni/MoN. The theoretical model of Ni cluster on the MoN (202) was applied to confirm the synergetic effect between Ni and MoN (**Figure** **5**a). Gibbs free energy diagram of hydrogen adsorption at different sites were exhibited in Figure S11 (Supporting Information), demonstrating the specific site (Figure 5a) on the interface between Ni and MoN possessed the smallest Δ*G*_H_* value of −0.19 eV, smaller than those of (202) MoN (−0.39 eV), and Ni cluster (−0.34 eV), and closed to that of Pt (−0.09 eV),^[^ ^37^ ^]^ as shown in Figure 5b.

Moreover, the density of state (Dos) and (d) band structure were employed to confirm the metallic characteristics of MoN, which could elevate the electron transfer from the current collector to catalysts. As shown in Figure 5c,d, the more occupations near the Fermi level and the narrow band gap closed to zero indicated that the metallic MoN possessed excellent conductivity,^[^ ^38^ ^]^ which was consisting with result of EIS (Figure 4c). In addition, the Atomic force microscope (AFM) and KPFM were used to detect the morphology and according potential distribution of Ni/MoN microrod. As shown in Figure 5e, the morphology of Ni/MoN microrod with diameter of 1.3 µm was consisted with the result of SEM image (Figure 1h). The rough surface with height distribution of ≈79 nm was due to the Ni nanoparticles embedded into MoN microrod. However, the potential distribution in Figure 5f presented the little difference between Ni nanoparticles and MoN microrod, suggesting the little difference of work function of Ni and metallic MoN, further proofing the similar Fermi level between Ni and metallic MoN. The aforementioned results confirmed the high HER catalytic activity was attributed to the interfacial catalytic sites of Ni/MoN and metallic characteristics of MoN.

**Figure 6:**
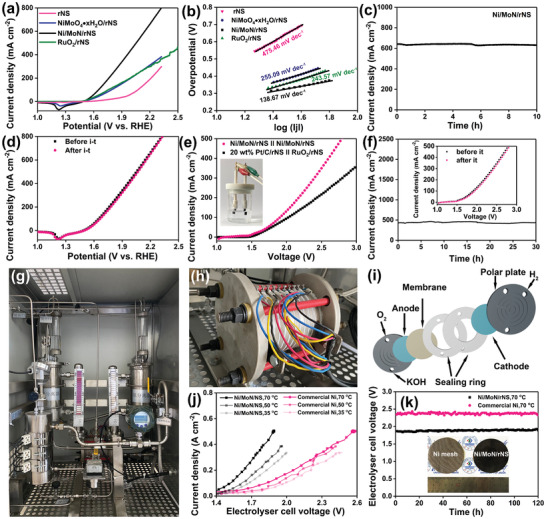
a) Polarization curves (back scanning) and b) corresponding Tafel plots of rNS, NiMoO_4_·xH_2_O/rNS, Ni/MoN/rNS, and RuO_2_/rNS in 1 m KOH for OER. c) The time dependent OER current density curve of Ni/MoN/rNS under an overpotential of 994 mV for 10 h and d) according polarization curves before and after *i*–*t* tests. e) Polarization curves for overall water splitting and f) the long‐term durability test of a water‐alkali electrolyzer of Ni/MoN/rNS||Ni/MoN/rNS and 20 wt% Pt/C/rNS||RuO_2_/rNS (inset: Polarization curves for overall water splitting before and after *i*–*t*). g) Photograph of industrial water splitting equipment. h) Enlarged horizon of the industrial electrolyzer. i) Schematic diagram of the industrial electrolyzer. j) Polarization curves measured during water electrolysis adopting Ni/MoN/rNS as both an anode and a cathode. k) The electrolyzer cell voltage of the electrolyzer held at 460 mA cm^−2^ for 120 h at 70 °C in 30 wt% KOH solution (inset shows the photographs of commercial Ni mesh and Ni/MoN/rNS electrodes with size of 6 cm).

The OER catalytic performance of the rNS, NiMoO_4_·xH_2_O/rNS, Ni/MoN/rNS and commercial RuO_2_ loaded on rNS (RuO_2_/rNS) were investigated by a typical three‐electrode cell. Juging from **Figure** **6**a, the Ni/MoN/rNS displayed the earliest onset and the fastest growth of the current with the applied potential when compared to rNS, NiMoO_4_·xH_2_O/rNF and RuO_2_/rNS. Ni/MoN/rNS only needed smallest overpotential (533 mV) to obtain a large current density of 200 mA cm^−2^, which was lower than those of rNS (979 mV), NiMoO_4_·xH_2_O/rNF (744 mV) and RuO_2_/rNS (738 mV). Ni/MoN/rNS exhibited the lowest Tafel slope of 138.7 mV dec^−1^, which was closed to that of RuO_2_/rNS (243.57 mV dec^−1^) and smaller than those of NiMoO_4_·xH_2_O/rNS (255.09 mV dec^−1^), rNS (475.46 mV dec^−1^), as shown in Figure 6b. Obviously, the R_ct_ of Ni/MoN/rNS was 2 Ω at 300 mV, which was far less than those of rNS (>600 Ω) and NiMoO_4_·xH_2_O/rNS (6 Ω), showing that the Ni/MoN/rNS possessed a fast electrocatalytic kinetic ability. Moreover, the electrochemical double‐layer capacitance (C_dl_) was performed to assess the electrochemically effective surface area (ECSA) of Ni/MoN/rNS, as shown in Figures S12 and S13 (Supporting Information). It was observed that Ni/MoN/rNS showed a high C_dl_ value of 10.8 mF cm^−2^, which was larger than those of rNS (0.7 mF cm^−2^) and NiMoO_4_·xH_2_O/rNS (6.6 mF cm^−2^), implying the more catalytic active sites of Ni/MoN/rNS. For comparing with others non‐noble‐metal electrocatalysts, an overview of overpotential for delivering 50 mA cm^−2^ in literatures were collected in Figure S14 and Table S3 (Supporting Information). To probe the durability of Ni/MoN/rNS, *i*–*t* test was carried out with an overpotential of 994 mV for 10 h (Figure 6c). As a result, the unchanged current density implied the outstanding durability of Ni/MoN/rNS during OER process. In addition, the polarization curve after stability test depicted negligible change compared with initial curve, signifying the retained OER activity of Ni/MoN/rNS (Figure 6d). Moreover, the unchanged morphology after durability test verified the outstanding stability during OER process (Figure S15, Supporting Information). After HER, there was no other peak in XPS spectra of NiMoN/rNS compared with that of present NiMoN/rNS as shown in Figure S16 (Supporting Information), indicated that no new phase of Ni(OH)_2_ was formed during HER process, which was also verified by FT‐IR Spectrometer (FT‐IR, Figure S17, Supporting Information) and Raman test (Figure S18, Supporting Information). After OER, the intensity of the peaks for Ni^0^ vanished, and nickel on the surface of the catalyst was mainly presented in Ni (II) state, but Ni (III) state appeared,^[^ ^39^ ^]^ indicating surface oxidation and formation of NiOOH on the surface of the NiMoN/rNS also processed during the HER as many Ni‐based metal nitride do.^[^ ^40^ ^]^ Moreover, the O 1s spectra of the electrodes showed hydroxyl was appeared after OER process apart pristine defect oxygen. These results suggested that the surface of the post‐OER electrode was mainly covered with an NiOOH layer and molybdenum oxides after OER.^[^ ^41^ ^]^ For pristine NiMoN/rNS, the intensity of three Raman peaks at 800–1000 cm^−1^ assigned to the Mo–O–Ni stretching vibration and the peak at 355 cm^−1^ assigned to Mo–O–Mo vibration.^[^ ^42^ ^]^ After OER process, the defined band at 554 cm^−1^ appeared that belong to thin layer NiOOH on NiMoN/rNS.^[^ ^43^ ^]^ Furthermore, the new peak at 900 cm^−1^ was assigned to MoO_4_^2−^ in alkaline solution, which originated from dissolution of Mo species.^[^ ^44^ ^]^ The characteristic peaks of NiOOH appeared in the FT‐IR spectrum (Figure S17, Supporting Information), which confirmed the formation of NiOOH.^[^ ^43^ ^]^

Due to excellent performance of Ni/MoN/rNS for HER and OER in 1 m KOH, the Ni/MoN/rNS was integrated as both anode and cathode to construct water‐alkali electrolyzer for overall water splitting (Figure 6e). To deliver a current density of 300 mA cm^−2^, Ni/MoN/rNS||Ni/MoN/rNS merely required 2.4 V in 1 m KOH, which was a little bit lower than that of (−) 20 wt% Pt/C/rNS||RuO_2_/rNS (+). The durability of Ni/MoN/rNS||Ni/MoN/rNS for overall water splitting was also experimented and was stated in Figure 6f. The catalytic current of cell for overall water splitting exhibited an ignore variation after 30 h *i*–*t* measure (≈500 mA cm^−2^), representing the excellent durability.

In our work, the Ni/MoN/rNS could be prepared in a large size for industrialization by virtue of a step hydrothermal followed by underfocus laser heating method (Figure 1b). Electrolysis of water for hydrogen was a widely used method of hydrogen production in industrial level and the according evaluation system as shown in Figure 6g. Alkaline electrolyzer was series unipolar pressure filter type structure, constituted by dozens of electrolysis chambers (Figure 6h). Each electrolysis chamber was consisted of anode polar plate, anode catalyst (oxygen production), membrane, sealing rings, cathode catalyst (hydrogen generation), and cathode polar plate (Figure 6i). To assess the performance of catalysts on industrial scale, Ni/MoN/rNS as both anode and cathode were employed to implement in industrial hydrogen generation systems (Figure S19, Supporting Information). Under various applied cell potentials, the current densities of the electrolyzer with the Ni/MoN/rNS electrodes were higher than the electrolyzer with commercial Ni electrodes. Even at identical current density, Ni/MoN/rNS also exhibited a lower cell voltage of 1.91 V compared with commercial Ni mesh electrodes (2.37 V). In addition, the current density was gradually increased with increased temperature from 35 to 50 and 70 °C (Figure 6j). No appreciable increase in the cell voltage during 120 h continuous operation with identical current density of 460 mA cm^−2^ was detected (Figure 6k), indicating the perfect stability of Ni/MoN/rNS in industrial electrolytic cell, which had possibly potential industrial applications. H_2_ gas was collected by a drainage method with output current of 8.5 A and the calculated Faraday efficiency was 82%, indicating its high conversion efficiency (The working energy efficiency of the alkaline electrolyte electrolyzer was usually 60–80%),^[^ ^45^ ^]^ as shown in Figure S20 (Supporting Information). There were two reasons for achievement of high current stability. First, Ni sheet was set as excellent conductive support materials, which promoted the electric transfer between Ni/MoN electrode and the power supply. Theoretically and practically, 3D microrods array structure of Ni/MoN/rNS with high electrochemically effective surface area provided more active sites, which could together promote the realization of high current density current. Second, laser roughening treatment was also helpful to the growth of nano‐materials and enhance the bonding force between Ni substrate and nano‐materials. Metallic MoN showed excellent conductivity with the same Ni, which showed excellent the electron transfer ability. 3D microrods structure of Ni/MoN/rNS did not be destroyed after high current density *i*–*t* test, indicating excellent resistance to lye corrosion.

In order to illustrate the universality of underfocus laser heating method, the precursor NiMoO_4_·xH_2_O/rNS were also treated by laser in other atmospheres, such as Ar, H_2_, CH_4_, and H_2_S. The according morphologies and structures were characterized by SEM images in Figure S21 (Supporting Information) and XRD pattern in Figure S22 (Supporting Information). Figure S21a (Supporting Information) showed the similar morphology of NiMoO_4_·xH_2_O/rNS‐Ar with NiMoO_4_·xH_2_O. The XRD pattern in Figure S15a (Supporting Information) illustrated that the NiMoO_4_·xH_2_O lost the crystal water and formed the crystalline NiMoO_4_. The NiMoO_4_·xH_2_O/rNS‐H_2_, NiMoO_4_·xH_2_O/rNS‐CH_4_, and NiMoO_4_·xH_2_O/rNS‐H_2_S all showed the porous microrods (Figure S21b–d, Supporting Information), but were successfully transformed into MoNi_4_/MoO_2_, Ni/Mo_2_C/MoO_2_, Ni_2_S_3_/Mo_7_S_8_/MoO_2_, respectively, confirmed by XRD pattern in Figure S22b–d (Supporting Information). The obtained NiMoO_4_·xH_2_O/rNS‐NH_3_ and NiMoO_4_·xH_2_O/rNS‐CH_4_ possessed small overpotentials of 105 and 111 mV at current density of 50 mA cm^−2^, which was smaller than those of NiMoO_4_·xH_2_O/rNS‐H_2_ (214 mV), NiMoO_4_·xH_2_O/rNS‐H_2_S (200 mV), and NiMoO_4_·xH_2_O/rNS‐Ar (169 mV), as shown in Figure S23a (Supporting Information), which also showed excellent HER stability, as shown in Figure S23b (Supporting Information). These results showed that the underfocus laser heating enabled the synthesis of various efficient electrodes, which were possible to be employed in other fields.

## 3 Conclusion

In summary, we reported a general and efficient method to fabricate various transition metal compound electrocatalysts by underfocus laser heating at different atmosphere, such as Ni/MoN/rNS, MoNi_4_/MoO_2_, Ni/Mo_2_C/MoO_2_, and Ni_2_S_3_/Mo_7_S_8_/MoO_2_. Among them, the Ni nanoparticles embedded metallic MoN microrods uniformly distributed on rNS (Ni/MoN/rNS) were fabricated by underfocus laser heating of NiMoO_4_·xH_2_O microrods with the conditions of ammonia atmosphere, room temperature and ambient pressure. The laser preparation mechanism of focus and underfocus modes confirmed the laser induced stress and local high temperature, which controllably and rapidly prepared the patterned and large size electrodes. The Ni/MoN/rNS with the excellent HER performance was attributed to the 3D structure of microrods array, high electrical conductivity of metallic MoN and the synergetic effect between Ni and MoN. The constructed two electrode system (−) Ni/MoN/rNS||Ni/MoN/rNS (+) showed superstability for overall water splitting with a high current density of 500 mA cm^−2^ for 30 h, which was superior to that of 20 wt% (−) Pt/C/rNS||RuO_2_/rNS (+). Remarkably, when using industrial electrolytic cell to evaluate water splitting activity in alkaline condition, the as‐synthesized Ni/MoN/rNS still afforded high activity and outstanding stability in hydrogen production (460 mA cm^−2^ for 120 h).

## Conflict of Interest

The authors declare no conflict of interest.

## Supporting information

Supporting information

## Data Availability

Research data are not shared.
