# Supplementary material for: Underfocus Laser Induced Ni Nanoparticles Embedded Metallic MoN Microrods as Patterned Electrode for Efficient Overall Water Splitting
Source: Adv Sci (Weinh). 2022 Feb 3;9(10):2105869. doi: 10.1002/advs.202105869 (PMC8981903; doi:10.1002/advs.202105869)
Supplement: Supplementary file 1 — Supporting information [file ADVS-9-2105869-s001.pdf]

ADVANCED SCIENCE
Open Access

Supporting Information

for *Adv. Sci.*, DOI 10.1002/advs.202105869

Underfocus Laser Induced Ni Nanoparticles Embedded Metallic MoN Microrods as Patterned Electrode for Efficient Overall Water Splitting

Yuke Chen, Yijie Wang, Jiayuan Yu, Guowei Xiong, Hongsen Niu, Yang Li, Dehui Sun, Xiaoli Zhang, Hong Liu and Weijia Zhou**

ADVANCED SCIENCE
Open Access

Supporting Information

for *Adv. Sci.,* DOI: 10.1002/advs.202105869

Underfocus Laser Induced Ni Nanoparticles Embedded Metallic MoN Microrods as Patterned Electrode for Efficient Overall Water Splitting

Yuke Chen, Yijie Wang, Jiayuan Yu, Guowei Xiong, Hongsen Niu, Yang Li, Dehui Sun, Xiaoli Zhang, Hong Liu, Weijia Zhou**

Underfocus Laser Induced Ni Nanoparticles Embedded Metallic MoN Microrods as Patterned Electrode for Efficient Overall Water Splitting

Yuke Chen, Yijie Wang, Jiayuan Yu, Guowei Xiong, Hongsen Niu, Yang Li, Dehui Sun, Xiaoli Zhang, Hong Liu, Weijia Zhou**

Y. Chen, Y. Wang, J. Yu, G. Xiong, Dr. D. Sun, Dr. H. Liu, Dr. W. Zhou
Collaborative Innovation Center of Technology and Equipment for Biological Diagnosis and Therapy in Universities of Shandong, Institute for Advanced Interdisciplinary Research (iAIR), University of Jinan, Jinan, 250022, P. R. China
E-mail: ifc_zhouwj@ujn.edu.cn (W. Zhou) and hongliu@sdu.edu.cn (H. Liu).
H. Niu, Dr. Y. Li
School of Information Science and Engineering, Shandong Provincial Key Laboratory of Network Based Intelligent Computing, University of Jinan, Jinan 250022, P. R. China
X. Zhang
School of Materials Science and Engineering, Zhengzhou University, Zhengzhou, 450001, China
H. Liu
State Key Laboratory of Crystal Materials, Shandong University, Jinan, 250100, P. R. China
E-mail: hongliu@sdu.edu.cn (H. Liu).

Keywords: Underfocus laser heating, Ni/MoN microrod, Large current density, Superstability, Industrial water-splitting electrolyser

Experimental Procedures

1 Materials

Ni sheet with thickness of 0.08 mm was obtained from the QingHe TengFeng Metal Co. Ltd.. Analytical grade ammonium molybdate tetrahydrate ($(NH_4)_6Mo_7O_{24}\cdot4H_2O$), sodium oxalate ($Na_2C_2O_4$), potassium hydroxide (KOH), hexamethylenetetramine ($C_6H_{12}N_4$), nickel nitride hexahydrate ($NiNO_3\cdot6H_2O$), absolute ethanol (≥ 99.7%), sulfuric acid (H_2SO_4, 98.3%), hydrochloric acid (HCl, 36.0~38.0%), 20 wt% Pt/C and ruthenium oxide (RuO_2) were acquired from Yantai Yuandong Fine Chemical Co.. Deionized water was provided by a Barnstead Nanopure Water System (18.2 MΩ·cm). Argon (Ar, 99.99 vt%), ammonia (NH_3, 99.99 vt %), methane (CH_4, 99.99 vt%), mixed gases of argon and hydrogen (10 vt%, H_2/Ar), and mixed gases of argon and hydrogen sulfide (20 vt%, H_2S/Ar) were purchased from Jinan XUSHENG Gases Co., Ltd..

2.1 Synthesis of Ni/MoN microrods on rNS

A fiber laser system (Universal LSF20D, Hgtech Laser, maximum output power 25 W) with a laser wavelength of 1064 nm and pulse duration of 100 ns was performed to induce the formation of Ni nanoparticles embedded metallic MoN microrods on the rNS. First, the commercial Ni sheet was roughened by infocus laser ablation with a scan rate of 1000 mm s^{-1}, output power of 12.5 W, and line spacing of 5 μm in linear scan mode. Then rNS was washed orderly with 1 M hydrochloric acid solution, alcohol, and deionized water for 30 min in order to remove organic matter and impurities existed on the surface of rNS. Then, 0.96 g of $Ni(NO_3)_2\cdot6H_2O$ and 0.8 g of $(NH_4)_6Mo_7O_{24}\cdot4H_2O$ were dissolved into 80 mL deionized water, and the above aqueous solution was added into a 100 mL Teflon autoclave. Next, the obtained rNS was set perpendicularly into above Teflon autoclave, then kept at 150 °C for 6 h. The $NiMoO_4\cdot xH_2O$ microrods grown on rNS ($NiMoO_4\cdot xH_2O$/rNS) was acquired when the autoclave cooled down to room temperature naturally. Lastly, the $NiMoO_4\cdot xH_2O$/rNS was placed in a round quartz glass reactor. The NH_3 gas with flow rate of 50 sccm was used as N source and reducing atmosphere. A fiber laser system (output power of 25 W, scan rate 1000 mm s^{-1}, line spacing of 5 μm) was employed to write regionally

on the $NiMoO_4{\cdot}xH_2O$/rNS. The UL was adjusted to 2 cm to generate an underfocus mode, then, the Ni/MoN/rNS was obtained. Furthermore, the different atmospheres (Ar, CH_4, H_2S, and H_2) with UL (2 cm) were also carried out at the same reaction condition to synthesize different samples.

2.2. Synthesis of MoN microrods

MoN microrods was synthesized by placing the Ni/MoN microrods (scraped from Ni/MoN/rNS) into 1 M H_2SO_4 solution for 24 hours under room temperature, subsequently washed by absolute ethyl alcohol and deionized water for several times.

2.3 Synthesis of Ni nanorods

The $NiC_2O_4{\cdot}2H_2O$ nanorods were synthesized by a room temperature stirring route. Typically, a solution of $Ni(NO_3)_2{\cdot}6H_2O$ (0.15 g) in 20 mL deionized water and 5 mL absolute ethyl alcohol was added to a solution of $Na_2C_2O_4$ (0.134 g), $C_6H_{12}N_4$ (0.14 g) in 20 mL deionized water and 5 mL absolute ethyl alcohol and mixed to obtain a stable solution, and then stirred vigorously at room temperature for 4 h. Finally, the obtained green samples were washed using deionized water and ethanol to remove the impurities, and dried by freezer dryer for 12 hours. The porous Ni nanorods were synthesized via a calcining process of $NiC_2O_4{\cdot}2H_2O$ nanorods. The pyrolysis temperature was set at 340 °C. In a typical process, the precursor of $NiC_2O_4{\cdot}2H_2O$ was placed in a tube furnace. Then, the furnace was heated to the temperature of 340 °C under Ar flow at a rate of 1 °C min^{-1} for 2 h.

3. Materials characterizations

Phase compositions of the materials were measured using a ARL Equinox 3000X (ThermoFisher) X-ray diffractometer (XRD) with Cu Kα radiation (λ=0.15406 nm). Materials morphologies and crystalline structure were identified using a field emission scanning electron microscope (FESEM; HITACHI regulus 8100) and a transmission electron microscope (TEM; Thermo Fischer Talos F200x Field Emission Electron Microscope) at an acceleration voltage of 200 kV. The elementary composition was performed by X-ray photoelectron spectroscopic (XPS) (Shimadzu, AXIS Ultra Supra). The reaction temperature was tested by Thermal Imaging Camera (Fotric 226) and Multimeter. Laser pressure of different focus degree of laser was

tested by Fine electronic balance instrument (SECURA213-1CN). Static contact angle measurements were performed with a JY-PHb apparatus made by Chengde Yote Instrument Co., Ltd., at room temperature in ambient air with 1.5 μL droplets of deionized water. ICP-AES analysis was measured on Model ARCOS FHS12 instrument. Fourier transform infrared spectrum (FT-IR) was collected on a Nicolet Avatar 360 infrared spectrometer using Attenuated Total Reflection (ATR) mode. Raman spectra were collected using a WITec CRM-200 fluorescence/Raman confocal imaging microscope coupled to a Princeton Instruments IsoPlane spectrometer, with a 532 nm excitation laser. The height of sample was detected by Atomic force microscope (AFM) measurements and (New Dimension icon, Bruker). The potential of sample was tested by Kelvin probe force microscopy (KPFM) measurements. The potential distribution of Ni/MoN microrod was detected by Kelvin probe force microscopy (KPFM) with the 50 nm Au film evaporated on Si plate as conductive basement and thus the Fermi level relative positions could be estimate.[1] The following formulas were used to voice the surface potential difference (SPD) between Ni, MoN, and the AFM tip

$$SPD_{Ni} = W_{Tip} – W_{Ni}$$

$$SPD_{MoN} = W_{Tip} – W_{MoN}$$

Where $W_{Tip,}$ W_{Ni}, and W_{MoN} were the work functions of the AFM Tip, Ni and MoN, respectively. Hence, the difference of Fermi level of Ni (E_{F-Ni}) and MoN (E_{F-MoN}) could be deduced by the following eqution

$$\Delta E_F = E_{F-Ni} – E_{F-MoN} = \Delta SPD = SPD_{Ni} – SPD_{MoN} = W_{MoN} – W_{Ni}$$

In order to obtain the work function of tip, Au film was used as the sample. As the standard sample, the work function of Au (W_{Au}) was a constant (5.1 eV) and the $SPD_{Au} = W_{Tip} – W_{Au}$ = 0.17 V, so the W_{Tip} is 5.27 eV. The SPD_{Ni} and SPD_{MoN} were 0.245 V and 0.232 V read from potential image, respectively. Therefore, the work function of W_{Ni} and W_{MoN} could be calculated to be 5.025 eV and 5.038 eV, respectively.

4. Electrochemical measurements

Electrochemical measurements were performed on an electrochemical workstation

(CHI 760E, CH Instruments, Inc.) with a standard three-electrode system in 1.0 M KOH electrolyte. Typically, a Hg/HgO electrode and a graphite rod electrode as reference and counter electrodes, respectively. The according potentials were calibrated to a reversible hydrogen electrode (RHE) by adding a value of (0.205 + 0.059 × pH). As for commercial 20 wt% Pt/C and RuO_2 power was well-dispersed in a diluted Nafion alcohol solution (500 μL of ethanol, 450 μL water, and 50 μL of Nafion) to form a homogeneous suspension. Then the above homogeneous suspension was dripped cast on the surface of the rNS at a catalyst loading of 0.5 mg cm^{-2}. The electrochemical activity of the samples (rNS, $NiMoO_4 \cdot xH_2O$/rNS, Ni/MoN/rNS, RuO_2/rNS and 20 wt% Pt/C/rNS electrodes) toward the HER and OER were researched via linear sweep voltammetry (LSV) curves with a potential sweep rate of 5 mV s^{-1}. Ni/MoN/rNS directly acted as cathode and anode was selected to construct the overall water splitting system. Electrochemical impedance spectroscopy (EIS) was carried out with an amplitude of 10 mV and a frequency range of 100 kHz to 0.01 Hz. The main arc in each EIS spectrum was fitted using a simplified Randles equivalent circuit, which consisted of a resistance (R_s) in series with a parallel arrangement of a charge transfer resistance (R_{ct}) and a constant phase element. Cyclic voltammetry was used to probe the electrochemical double-layer capacitance at non-faradaic potentials to estimate the effective electrode surface area. The current-time responses were monitored using chronoamperometric measurements at large current densities.

The industrial performance of Ni/MoN/rNS were tested under industrial conditions of 30 wt% KOH solution with a temperature of 70 ^{o}C, which was carried out by an electrolyser from the Cockerill jingli Hydrogen, China. The Ni/MoN/rNS disc electrodes with diameter of 6 cm were integrated as both anode and cathode, respectively. The mature Ni mesh as both anode and cathode used in industrial hydrogen production system was set as blank electrode. The current density of water splitting was simultaneously monitored at different temperatures.

5. Density Functional Theory (DFT)Calculation

All the spin-polarized computations were performed by using Vienna ab initio simulation package (VASP).[2, 3] The ion-electron interactions were described by the

projector augmented wave method[4] and the general gradient approximation in the Perdew–Burke–Ernzerhof (PBE) form was used.[5, 6] A Ni_{10} cluster supported on 2 × 4 MoN (202) slab were used as the models. During the structure relaxation, the convergence criterion was set to 0.03 eV/Å and 10^{-5} eV for the residual force and energy, respectively. Brillouin zones were sampled by a Monkhorst-Pack 2 × 3 × 1 k-point grid. To avoid the interaction between two periodic units, a vacuum space of 15 Å was employed.

The HER activity was evaluated by the free energy for hydrogen adsorption (ΔG_{H})[7], which was calculated as

$$\Delta G_{H} = \Delta E + \Delta E_{ZPE} - T\Delta S$$

where ΔE, E_{ZPE}, T and S is the reaction energy difference, zero-point energies, temperature and entropy, respectively.

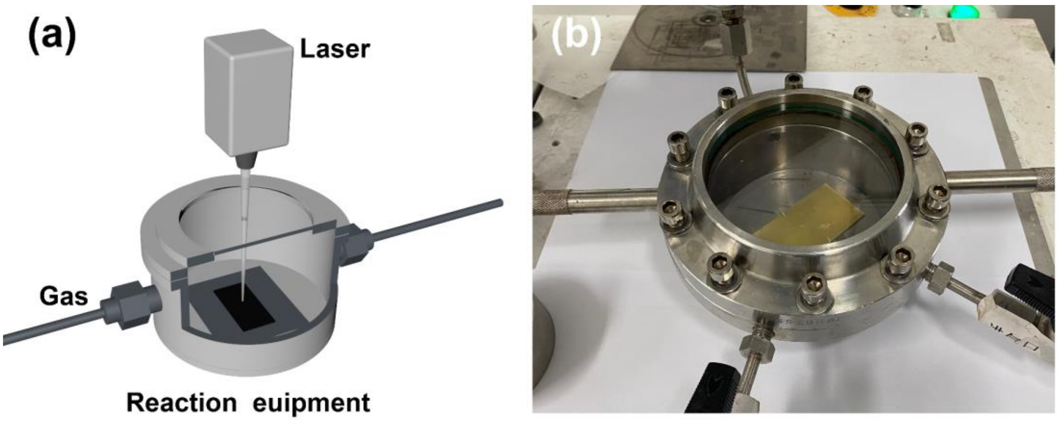

Figure S1. (a) Schematic diagram and (b) photograph of reaction chamber with 5 mm thick SiO_2 window, which was used to supplied a stable gas condition for laser heating reaction.

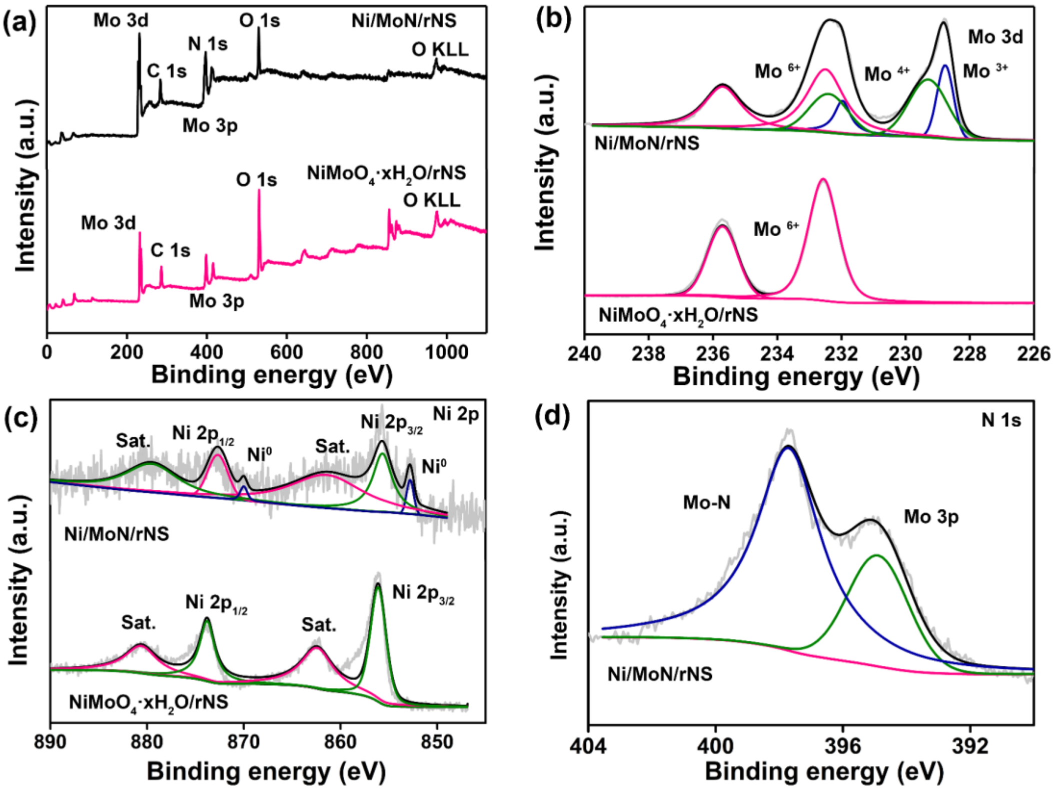

Figure S2. (a) Full spectrum X-ray photoelectron spectroscopy (XPS) spectra and high-resolution XPS spectra of (b) Mo 3d and (c) Ni 2p of $NiMoO_4{\cdot}xH_2O$/rNS and Ni/MoN/rNS (d) N 1s of Ni/MoN/rNS.

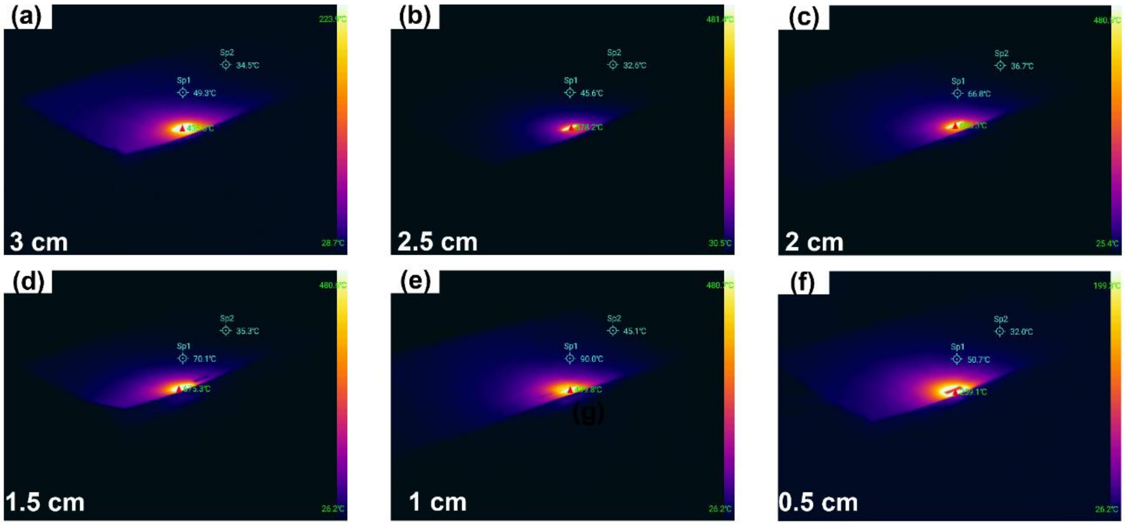

Figure S3. (a-f) Thermal imaging pictures of synthesized samples at different degree of underfocus of 3 cm, 2.5 cm. 2 cm, 1.5 cm, 1 cm and 0.5 cm, respectively.

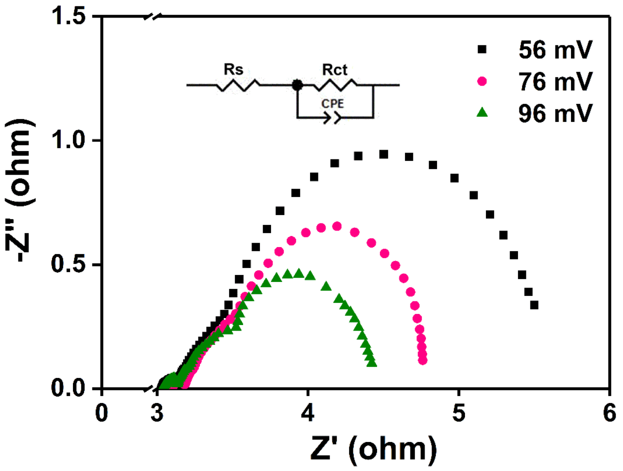

Figure S4. EIS Nyquist plots of Ni/MoN/rNS with overpotentials from 56, 76 to 96 mV.

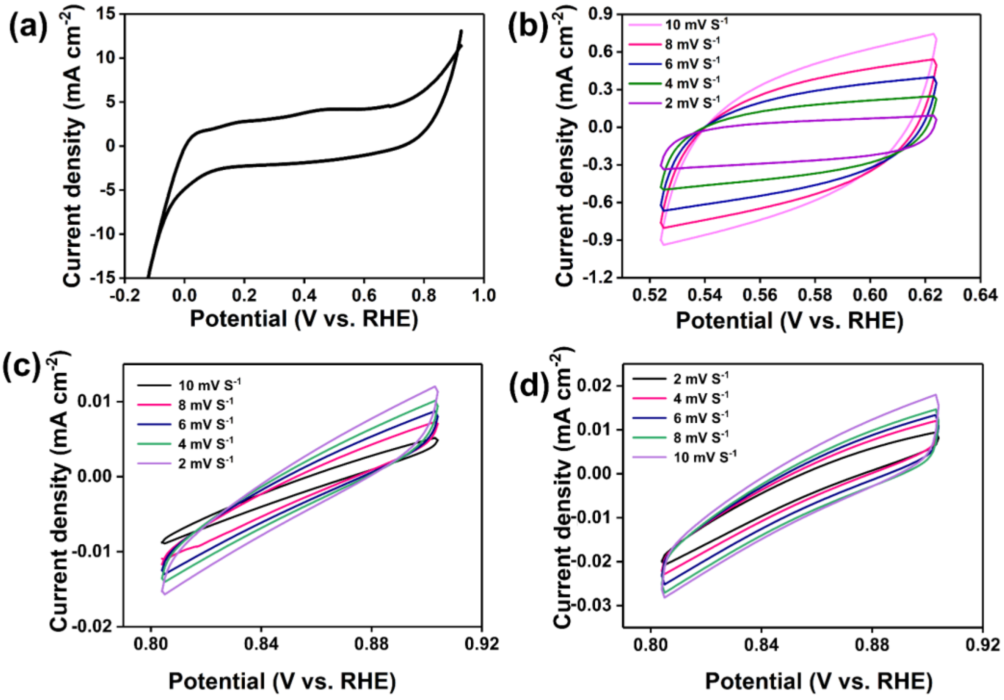

Figure S5. (a) The whole curve of Ni/MoN/rNS and cyclic voltammograms without faradaic reactions of (b) Ni/MoN/rNS, (c) rNS, and (d) $NiMoO_4 \cdot xH_2O$/rNS.

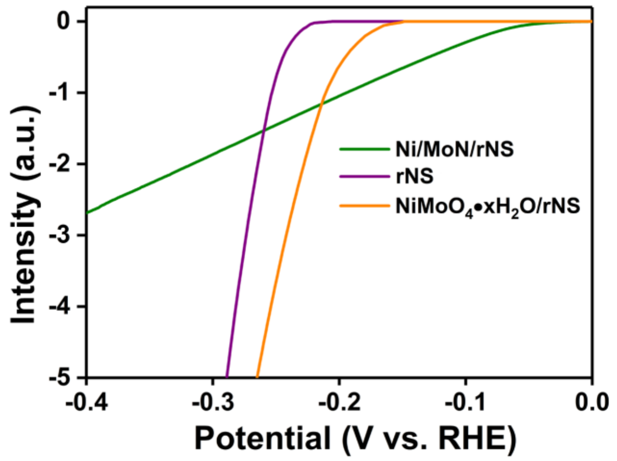

Figure S6. Polarization curves of Ni/MoN/rNS, rNS, and $NiMoO_4 \cdot xH_2O$/rNS normalized by the respective electrochemical surface area toward HER in 1 M KOH.

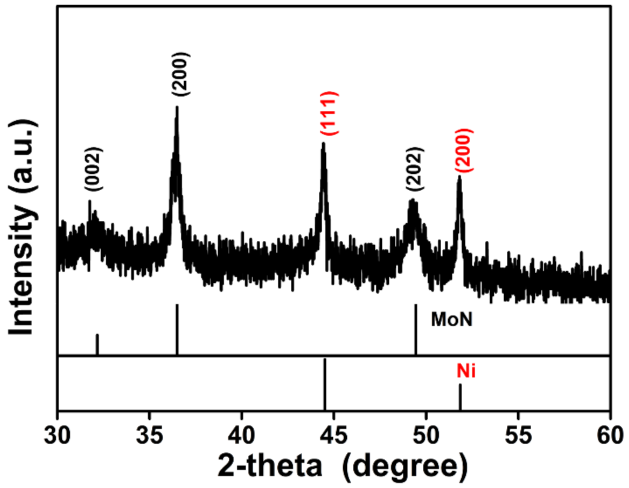

Figure S7. XRD pattern of Ni/MoN/rNS after it test for HER.

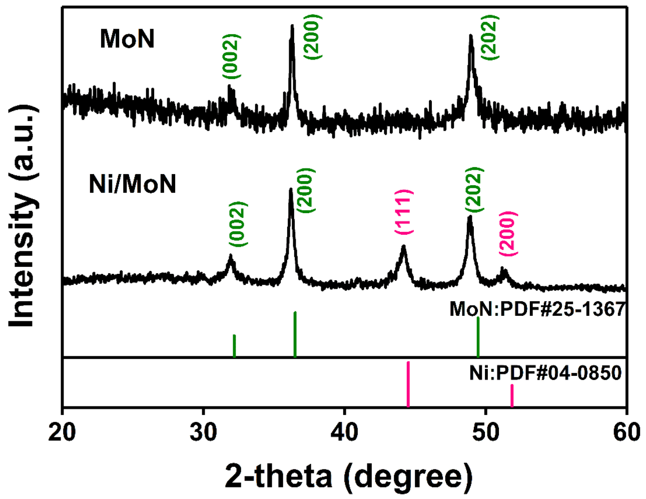

Figure S8. XRD pattern of Ni/MoN microrod and MoN microrods.

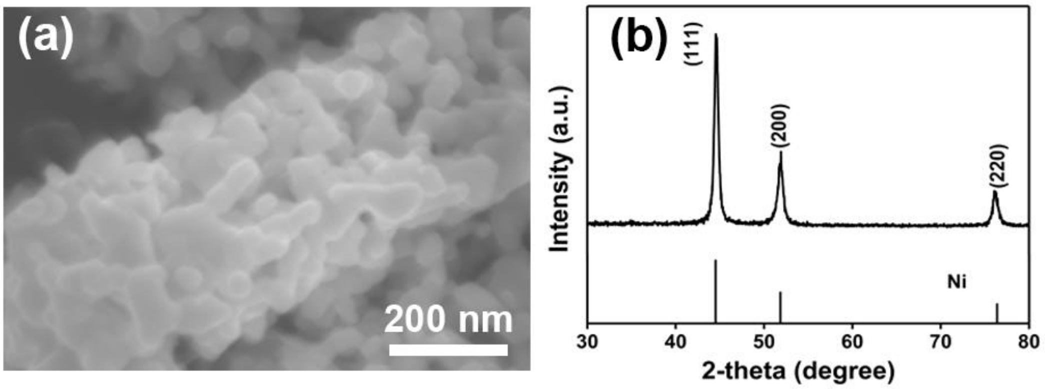

Figure S9. (a) SEM image and (b) XRD pattern of porous Ni nanorods.

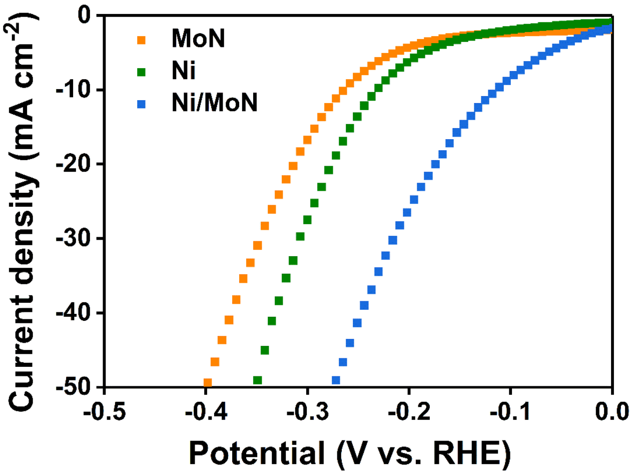

Figure S10. LSV curves of Ni/MoN, MoN, and Ni nanorods toward HER in 1 M KOH.

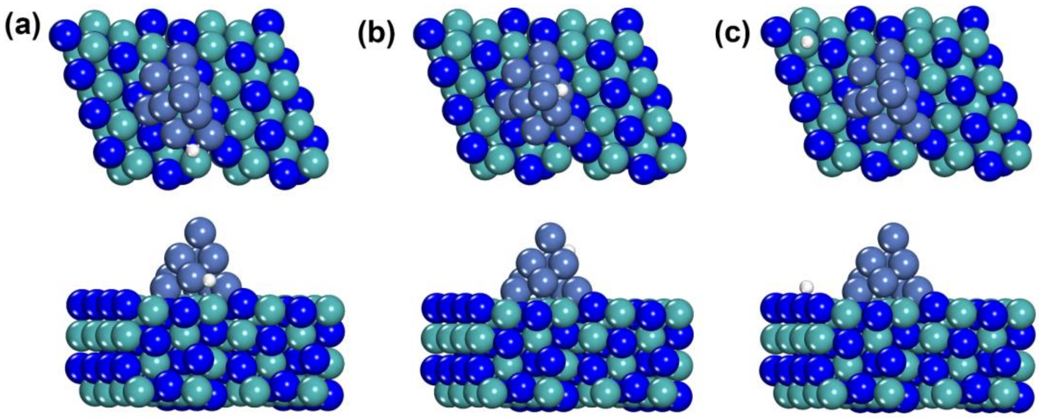

Figure S11. The ΔG_{H*} values of hydrogen adsorption on the different sites for (a) MoN-Ni interface, (b) Ni cluster and (c) MoN (202).

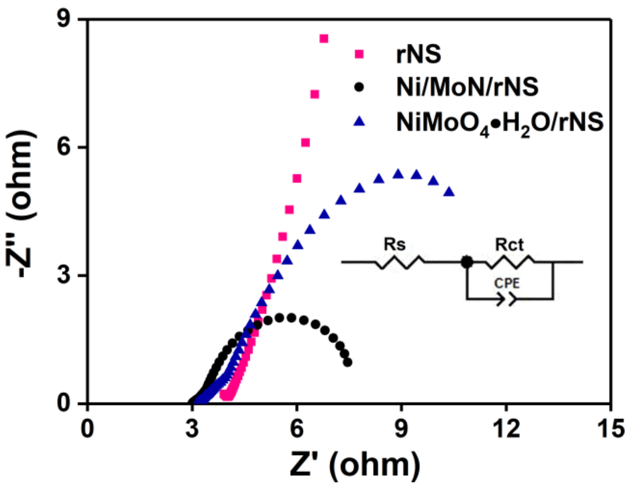

Figure S12. Nyquist plots with the overpotential of 300 mV (inset: the equivalent circuit) of rNS, $NiMoO_4 \cdot xH_2O$/rNS, Ni/MoN/rNS.

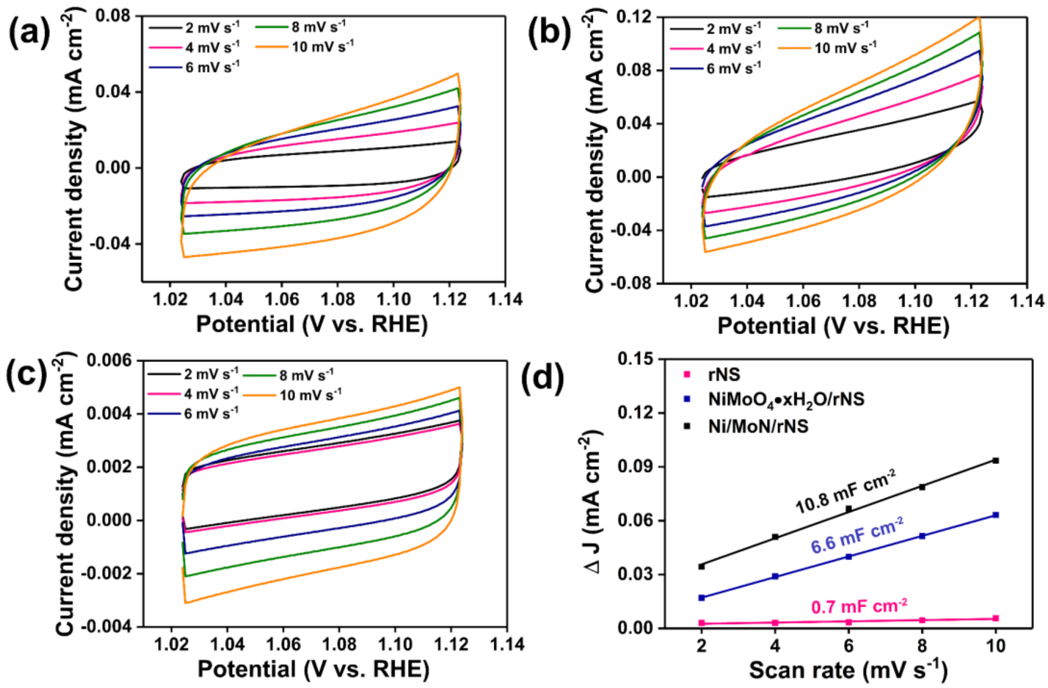

Figure S13. Cyclic voltammograms without faradaic reactions of (a) $NiMoO_4 \cdot xH_2O$/rNS, (b) Ni/MoN/rNS, and (c) roughened Ni Sheet for alkaline oxygen evolution. (d) Electrochemical area of rNS, $NiMoO_4 \cdot xH_2O$/rNS, Ni/MoN/rNS under OER conditions.

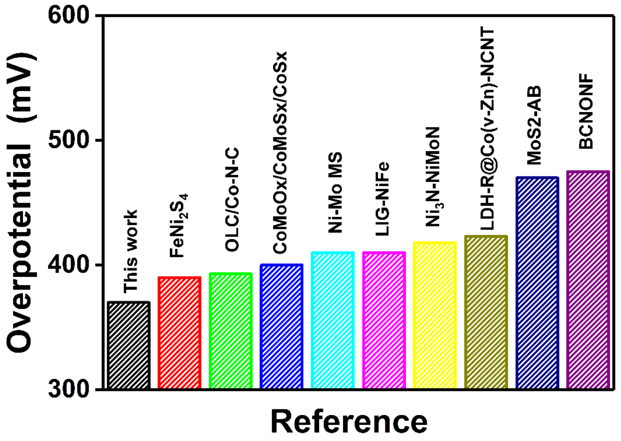

Figure S14. Comparison of the required voltage at a current density of 50 mA cm^{-2} for the Ni/MoN/rNS with reported state-of-the-art noble metal free catalysts.

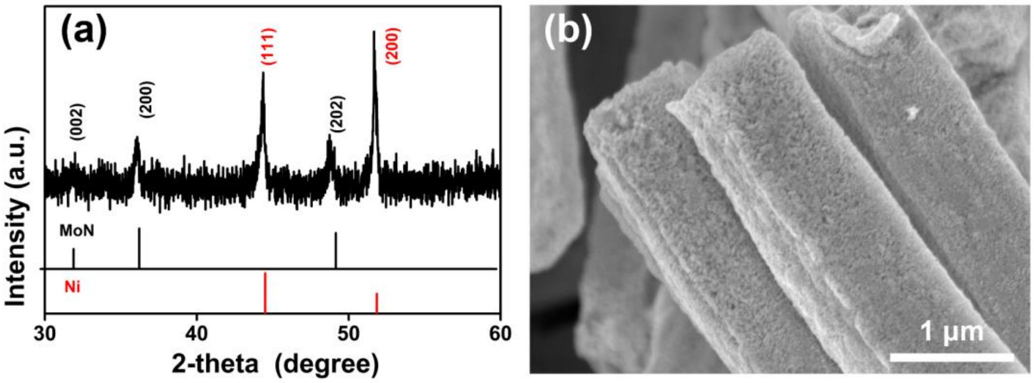

Figure S15. XRD pattern and SEM image of Ni/MoN/rNS after it test for OER.

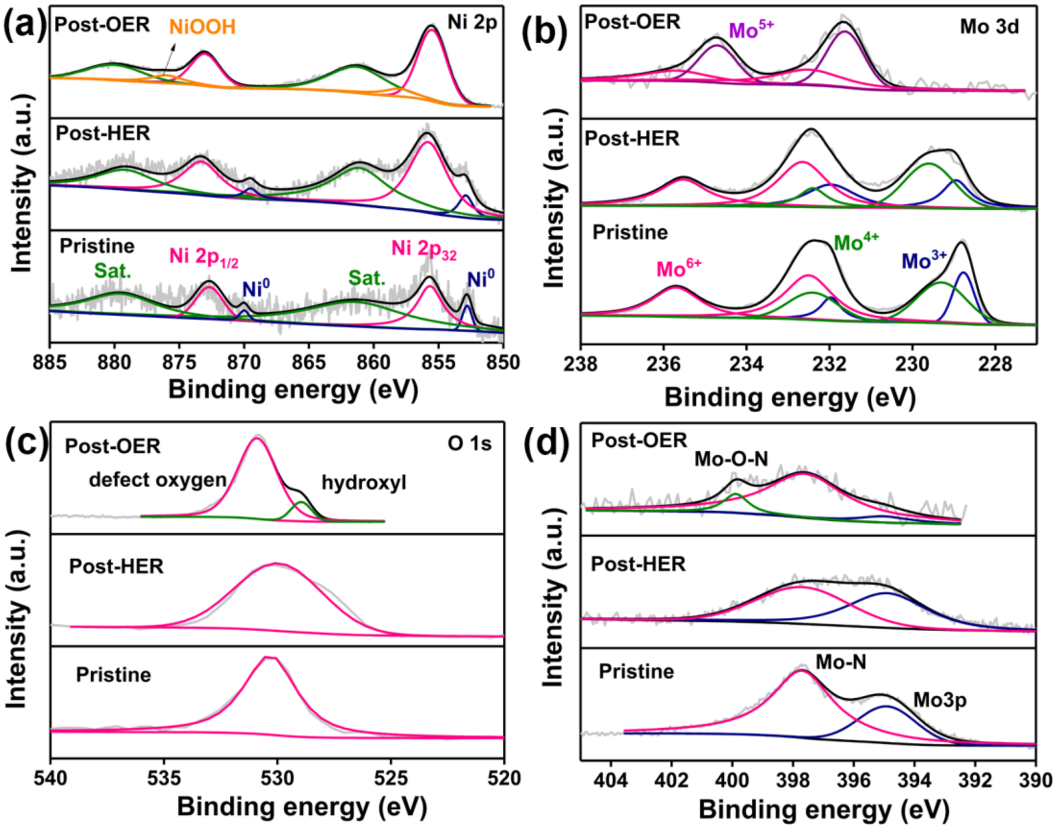

Figure S16. XPS spectra of Ni 2p (a), Mo 3d (b), O 1s (c) and N 1s (d) for Ni/MoN/rNS electrodes before and after HER and OER test.

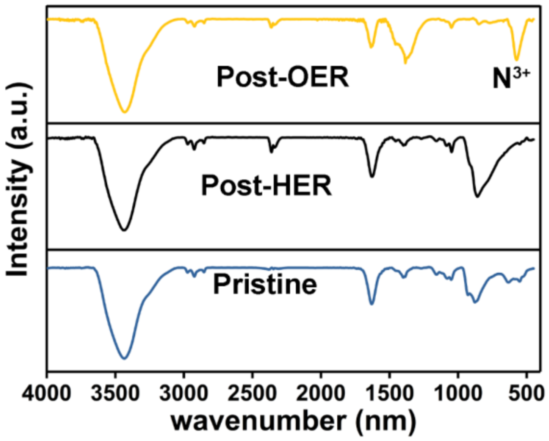

Figure S17. FT-IR spectrum of Ni/MoN/rNS before and after HER and OER test.

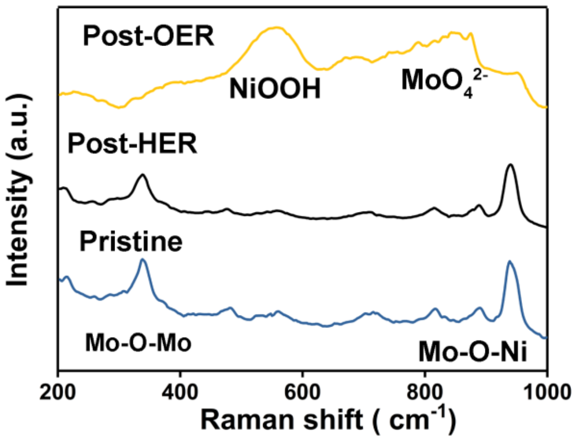

Figure S18. Raman spectra of Ni/MoN/rNS before and after i-t test.

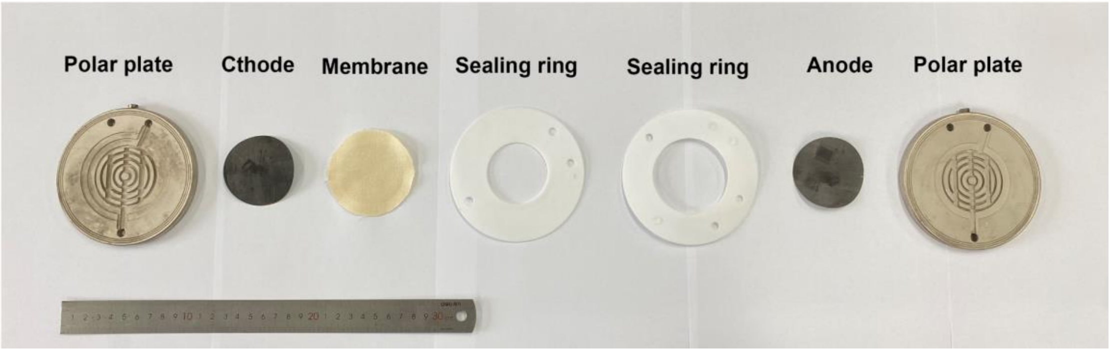

Figure S19. Stack structure of the electrolysis cell used in the machine testing.

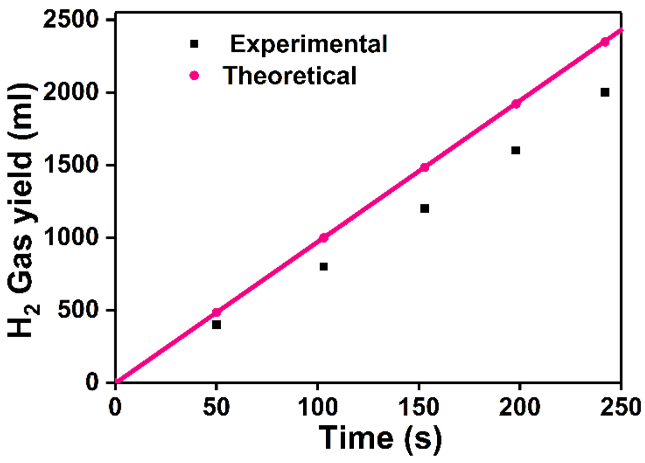

Figure S20. Gas evolution during the HER under industrial conditions.

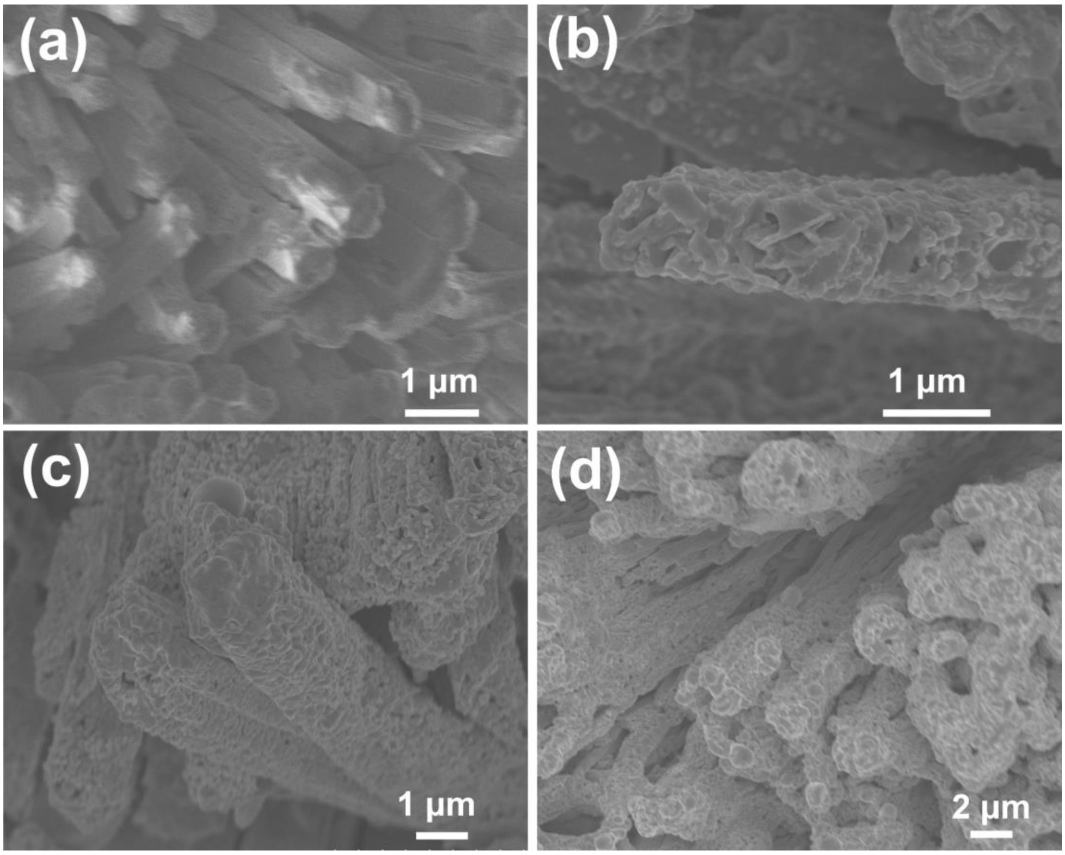

Figure S21. SEM images of the $NiMoO_4 \cdot xH_2O$/rNS treated by laser under atmosphere of (a) Ar, (b) H_2, (c) CH_4, and (d) H_2S in the underfocus mode.

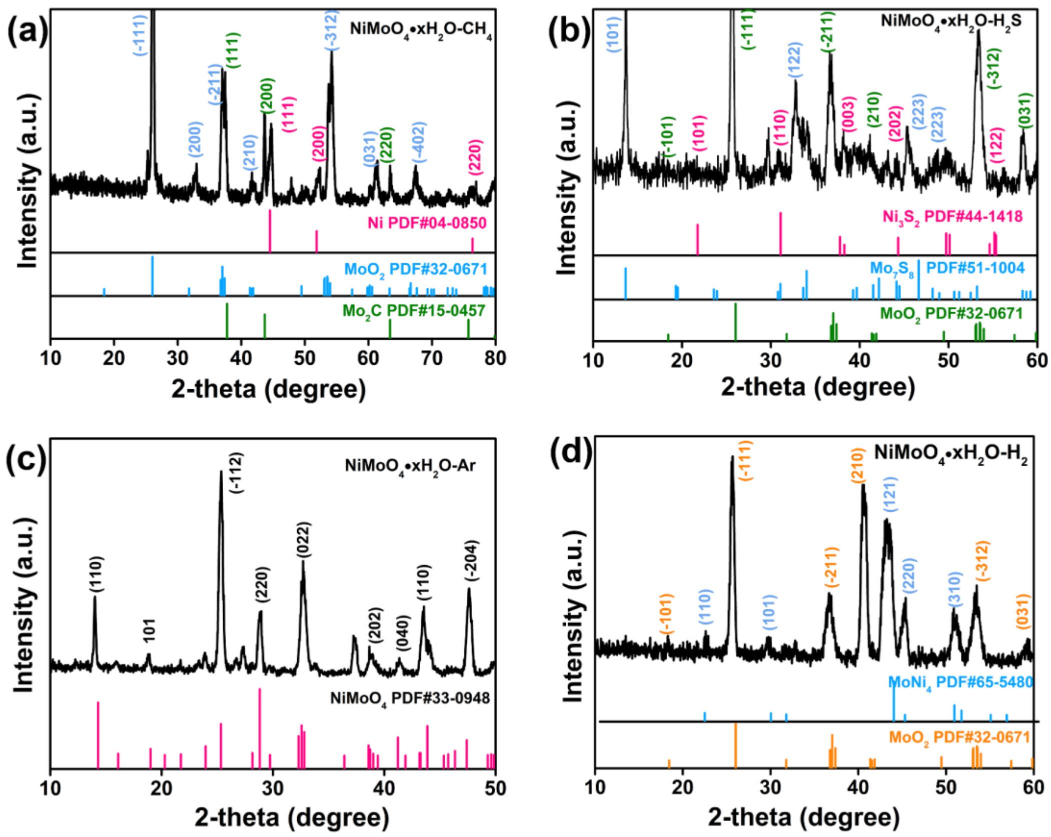

Figure S22. XRD patterns of sample treated at different atmosphere in the underfocus mode.

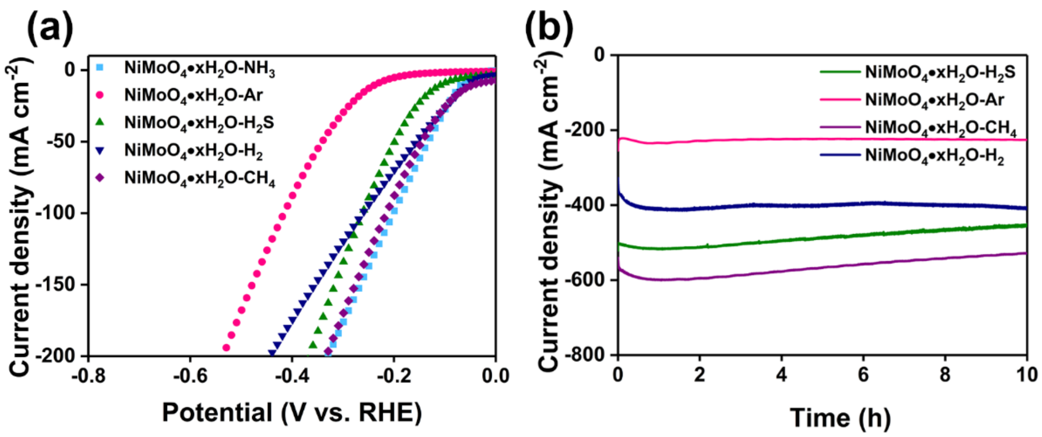

Figure S23. LSV and i-t curves of samples treated at different atmosphere gas in the underfocus mode for HER in 1 M KOH.

Table S1. ICP-AES result of Ni/MoN catalyst.

Sample	Ni (wt%)	Mo (wt%)	N (wt%)

Ni/MoN	44.91%	28.39%	

Table S2. Comparison of the HER activity of Ni/MoN/rNS with other non-noble-metal electrocatalysts.

electrocatalysts	η_{50} Overpotential(mV)	Electrolyte	Ref.
Ni/MoN/rNS	105 mV	1.0 M KOH	This work
MoO_3/Ni–NiO	143 mV	1.0 M KOH	[8]
$MnCo_2O_4$@Ni_2P/NF	120 mV	1.0 M KOH	[9]
Ni_2P/Ni@C	220 mV	1.0 M KOH	[10]
meso-Fe-MoS_2/$CoMo_2S_4$	192 mV	1.0 M KOH	[11]
FeOOH/Ni_3N	140 mV	1.0 M KOH	[12]
Mo-$NiCo_2O_4$/$Co_{5.47}N$/NF	172 mV	1.0 M KOH	[13]
O-CoP-2	145 mV	1.0 M KOH	[14]
Ni/$Ni(OH)_2$	114 mV	1.0 M KOH	[15]
V-CoP@a-CeO_2	138 mV	1.0 M KOH	[16]
NiMnOP	125 mV	1.0 M KOH	[17]
CdS@CoNiSx	215 mV	1.0 M KOH	[18]
NiMoS	128 mV	1.0 M KOH	[19]
WMo-NG	145 mV	1.0 M KOH	[20]
Mo-Co_9S_8@C	225 mV	1.0 M KOH	[21]
Ni_2P/$NiTe_2$	125 mV	1.0 M KOH	[22]
β-Mo_2C@NPCC	265 mV	1.0 M KOH	[23]
NFP/C-3	150 mV	1.0 M KOH	[24]
MoP/0.5CA-CDs1100	175 mV	1.0 M KOH	[25]
$NiTe_2$ NWs	190 mV	1.0 M KOH	[26]
$Ni_3Sn_2S_2$@Ni_3S_2 NFs	125 mV	1.0 M KOH	[27]

Table S3. Comparison of the OER activity of Ni/MoN/rNS with other non-noble-metal electrocatalysts.

electrocatalysts	η_{50} Overpotential(mV)	Electrolyte	Ref.
Ni/MoN/rNS	370 mV	1.0 M KOH	This work
LIG-NiFe	410 mV	1.0 M KOH	[28]
Ni–Mo MS	410 mV	1.0 M KOH	[29]
$FeNi_2S_4$	390 mV	1.0 M KOH	[30]
LDH-R@Co(v-Zn)-NCNT	423 mV	1.0 M KOH	[31]
OLC/Co-N-C	393 mV	1.0 M KOH	[32]
CoMoOx/CoMoSx/CoSx	400 mV	1.0 M KOH	[33]
Ni_3N-NiMoN	418 mV	1.0 M KOH	[34]
MoS_2-AB	470 mV	1.0 M KOH	[35]
BCNONF	475 mV	1.0 M KOH	[36]

Reference:

1. G. Wang, L. Li, W. Fan, R. Wang, S. Zhou, J.-T. Lü, L. Gan and T. Zhai, *Adv. Funct. Mater.*, 2018, **28**, 1800339.
2. G. Kresse and J. Furthmüller, *Phys. Rev. B*, 1996, **54**, 11169.
3. G. Kresse and D. Joubert, *Phys. Rev. B*, 1999, **59**, 1758-1775.
4. P. E. Blöchl, *Phys. Rev. B*, 1994, **50**, 17953-17979.
5. J. P. Perdew, J. Chevary, S. Vosko, K. A. Jackson, M. R. Pederson, D. Singh and C. Fiolhais, *Phys. Rev. B*, 1992, **46**, 6671-6687.
6. J. P. Perdew and Y. Wang, *Phys. Rev. B*, 1992, **45**, 13244.
7. J. K. Nørskov, T. Bligaard, A. Logadottir, J. R. Kitchin, J. G. Chen, S. Pandelov and U. Stimming, *J. Electrochem. Soc.*, 2005, **152**, J23.
8. X. Li, Y. Wang, J. Wang, Y. Da, J. Zhang, L. Li, C. Zhong, Y. Deng, X. Han and W. Hu, *Adv. Mater.*, 2020, **39**, 2003414.
9. J. Ge, W. Zhang, J. Tu, T. Xia, S. Chen and G. Xie, *small*,2020, 2001856.
10. X. Liu, W. Li, X. Zhao, Y. Liu, C. W. Nan and L. Fan, *Adv. Funct. Mater.*, 2019, **29**, 1901510.
11. Y. Guo, J. Tang, J. Henzie, B. Jiang, W. Xia, T. Chen, Y. Bando, Y.-M. Kang, M. S. A. Hossain Y. Sugahara and Y. Sugahara, *ACS nano*, 2020, **14**, 4141-4152.
12. J. Guan, C. Li, J. Zhao, Y. Yang, W. Zhou, Y. Wang and G.-R. Li, *Appl. Catal. B*, 2020, **269**, 118600.

13. W. Liu, L. Yu, R. Yin, X. Xu, J. Feng, X. Jiang, D. Zheng, X. Gao, X. Gao and W. Que, P. Ruan, F. Wu, W. Shi and X.Cao, *Small*, 2020, **16**, 1906775.
14. G. Zhou, M. Li, Y. Li, H. Dong, D. Sun, X. Liu, L. Xu, Z. Tian and Y. Tang, *Adv. Funct. Mater.*, 2020, **30**, 1905252.
15. L. Dai, Z. N. Chen, L. Li, P. Yin, Z. Liu and H. Zhang, *Adv. Mater.*, 2020, **32**, 1906915.
16. L. Yang, R. Liu and L. J. Jiao, *Adv. Funct. Mater.*, 2020, **30**, 1909618.
17. J. Balamurugan, T. T. Nguyen, V. Aravindan, N. H. Kim and J. Lee, *Nano Energy*, 2020, **69**, 104432.
18. F. Si, C. Tang, Q. Gao, F. Peng, S. Zhang, Y. Fang and S. Yang, *J. Mater. Chem. A*, 2020, **8**, 3083-3096.
19. C. Wang, X. Shao, J. Pan, J. Hu and X. Xu, 2020, *Appl. Catal. B*, **268**, 118435.
20. Y. Yang, Y. Qian, H. Li, Z. Zhang, Y. Mu, D. Do, B. Zhou, J. Dong, W. Yan, Q. Yong, L. Fang, R. Feng, J. Zhou, P. Zhang, J. Dong, G. Yu, Y. Liu, X. Zhang, and X. Fan, *Sci. Adv.*, 2020, **23**, eaba6586.
21. L. Wang, X. Duan, X. Liu, J. Gu, R. Si, Y. Qiu, Y. Qiu, D. Shi, F. Chen and X. Sun, *Adv. Energy Mater.*, 2020, **10**, 1903137.
22. Y. Li, X. Tan, H. Tan, H. Ren, S. Chen, W. Yang, S. C. Smith, C. J. E. Zhao and E. *Science*, 2020.
23. H. Yang, X. Chen, G. Hu, W.-T. Chen, S. J. Bradley, W. Zhang, G. Verma, T. Nann, D.-e. Jiang and P. Kruger, X. Wang, S. Telfer and S. Ma, *Chem. Sci.*,2020, **11**, 3523-3530.
24. Y. Li, B. Liu, H. Wang, X. Su, L. Gao, F. Zhou and G. Duan, *Sci. China Mater.*, 2018, **61**, 1575-1586.
25. H. Song, Y. Li, L. Shang, Z. Tang, T. Zhang and S. Lu, *Nano Energy*, 2020, 104730.
26. S. Anantharaj, S. Kundu and S. Noda, *J. Mater. Chem. A*, 2020, **8**, 4174-4192.
27. S.-Y. Lu, S. Li, M. Jin, J. Gao and Y. Zhang, *Appl. Catal. B*, 2020, **267**, 118675.
28. J. Zhang, C. Zhang, J. Sha, H. Fei, Y. Li and J. M. Tour, *ACS Appl. Mater. Interfaces*, 2017, **9**, 26840-26847.
29. M. Y. Gao, C. Yang, Q. B. Zhang, J. R. Zeng, X. T. Li, Y. X. Hua, C. Y. Xu and P. Dong, *Journal of Materials Chemistry A*, 2017, **5**, 5797-5805.
30. J. Jiang, Y.-J. Zhang, X.-J. Zhu, S. Lu, L.-L. Long and J.-J. Chen, *Nano Energy*, 2021, **81**, 105619.
31. W. Yang, Y. Bai, J. Ma, Z. Wang, W. Sun, J. Qiao, H. Cai and K. Sun, *J. Mater. Chem. A*, 2020, **8**, 25268-25274.
32. Z. Liang, N. Kong, C. Yang, W. Zhang, H. Zheng, H. Lin and R. Cao, *Angew. Chem. Int. Ed*, 2021, **60**, 12759-12764.
33. H. Xu, H. Shang, C. Wang, L. Jin, C. Chen, C. Wang and Y. Du, *Appl. Catal. B*, 2020, **265**, 118605.
34. A. Wu, Y. Xie, H. Ma, C. Tian, Y. Gu, H. Yan, X. Zhang, G. Yang and H. Fu, *Nano Energy*, 2018, **44**, 353-363.
35. L. Guo, Q. Liu, Y. Liu, Z. Chen, Y. Jiang, H. Jin, T. Zhou, J. Yang and Y. Liu, *Nano Energy*, 2022, **92**, 106707.

36. H. Li, B. Ren, W. Liu, L. Jing, R. Y. Tay, S. H. Tsang, L. Ricardez–Sandoval, A. Yu and E. H. T. Teo, *Nano Energy*, 2021, **88**, 106246.
